# 1,25-Dihydroxyvitamin D3 modulates adipogenesis of human adipose-derived mesenchymal stem cells dose-dependently

**DOI:** 10.1186/s12986-021-00561-4

**Published:** 2021-03-12

**Authors:** Amin Salehpour, Mehdi Hedayati, Farzad Shidfar, Asal Neshatbini Tehrani, Ali Asghar Farshad, Saeed Mohammadi

**Affiliations:** 1 Occupational Health Research Center, School of Public Health, Iran University of Medical Sciences, Tehran, Iran; 2 Cellular and Molecular Endocrine Research Center, Research Institute for Endocrine Sciences, Shahid Beheshti University of Medical Sciences, 2nd Floor, Number 24, Parvaneh Street, Yemen Street, Chamran Exp, Tehran, Iran; 3 Department of Nutrition, School of Paramedical, Ahvaz Jundishapur University of Medical Sciences, Ahvaz, Iran; 4 Department of Biostatics, School of Public Health, Iran University of Medical Sciences, Tehran, Iran

**Keywords:** 1,25-Dihydroxyvitamin D3, Obesity, Mesenchymal stem cells, Adipogenic differentiation

## Abstract

**Purpose:**

1,25-dihydroxyvitamin D3 may regulate adipogenesis in adipocytes in-vitro, but little is known about possible molecular mechanisms related to the inhibitory effect of 1,25-dihydroxyvitamin D3 on adipogenesis in humans҆ adipose tissue.

**Methodology:**

In this study, human adipose-derived mesenchymal stem cells (hASCs) were cultured for 14 days in adipogenic differentiation media containing concentrations of 1,25-dihydroxyvitamin D3 (10^−10^–10^−8^ M). The extent of adipogenic differentiation in ASCs was assessed by Oil Red O staining and quantitative polymerase chain reaction (PCR) to determine expression levels of key adipogenic markers.

**Results:**

Our results showed that vitamin D receptor (VDR), as a mediator of most actions of 1,25-dihydroxyvitamin D3, glucose trasporter-4 (GLUT4),and fatty acid binding protein-4 (FABP4) was expressed in vitamin D-treated hASCs. However, the protein level of these markers was lower than the control group. Treatment of human preadipocytes with 1,25-dihydroxyvitamin D3 significantly altered expression of adipogenic markers and triglyceride accumulation in a dose-dependent manner. 1,25-dihydroxyvitamin D3 at concentration of 10^−8^ M enhanced expression of sterol regulatory element-binding protein-1c (SREBP1c), CCAAT-enhancer-binding protein-β (C/EBPβ), a mitotic clonal expansion, peroxisome proliferator-activated receptor-gamma (PPARγ), fatty acid synthase (FASN), a marker of de novo lipogenesis,and lipoprotein lipase (LPL).

**Conclusion:**

Our findings revealed that 1,25-dihydroxyvitamin D3 may provoke adipocyte development in critical periods of adipogenesis at concentration of 10^−8^ M, thereby leading to a greater risk of obesity in adulthood and an augmented risk of obesity-related diseases including diabetes, cardiovascular diseases, and some cancers.

## Introduction

Obesity characterized by accumulation of exorbitant triglyceride in adipose tissue depending on hypertrophy and/or hyperplasia of adipocytes is one of the main public health concerns and it has been known as a key risk factor for progression of metabolic syndrome, type 2 diabetes mellitus, cardiovascular disease, cancer ,and reduction of life expectancy [1, 2]. Therefore, obesity is considered as a concern for the patients, policy-makers, and third-party payers [3, 4].

Vitamin D is one of the fat-soluble vitamins with exogenous and endogenous source in body. Ultraviolet B (UVB) irradiation in the skin leads to the increase in the level of vitamin D in body through conversion of 7-dehydrocholestrol into cholecalciferol (vitamin D3). Diet can provide the body’s requirement for vitamin D as well. For activating vitamin D thoroughly, it should be hydroxylated twice. Under reaction with 25-hyrodxylases, previtamin D is turned into 25-hidroxyvitamin D3 (25(OH)D), as circulating form of vitamin D. Then, 1,25-dihydroxy-vitamin D (1,25(OH)_2_D) as a bioactive form of vitamin D metabolite and activator of vitamin D receptor (VDR) is obtained from 25(OH)D through action of 1α-hydroxylase [5].

Convincing data have indicated a relationship between obesity and vitamin D [6–8]. Besides, the conventional role of vitamin D in systemic calcium homeostasis and bone metabolism, vitamin D endocrine system has various extra skeletal targets including adipocytes [6–8]. Interestingly, 1,25-dihydroxyvitamin D3 binds to VDR, acting as a pleiotropic endocrine hormone and influencing proliferation, differentiation, apoptosis, and gene expression.

Interaction of 1,25(OH)2D with nuclear VDR is responsible for transcription regulation of many genes, involving in regulation of cell proliferation and differentiation, immune function ,and metabolism in different types of cells [9, 10]. There is a large body of literature regarding provoking action of 1,25-dihydroxyvitamin D3 at low concentrations and its prohibiting and stimulating actions in differentiation and apoptosis, respectively at high concentrations [10–12]. Additionally, it seems that 25(OH) D could be involved in adipogenic differentiation of human preadipocytes, most likely through its conversion into 1,25(OH)2D [13].

Expression of VDR in adipocytes is the keystone for action of 1,25-dihydroxyvitamin D3 in adipose tissue and energy homeostasis [6–8, 14–16]. The previous studies have indicated expression of the genes encoding the enzymes converting or catalyzing vitamin D including cytochrome P450 enzymes of CYP27B1, CYP2R1, and CYP24 in adipocytes. Thus, local synthesis along with degradation of biologically active form of vitamin D could be happened in adipocytes [17–19]. Moreover, many vitamin D metabolizing enzymes are also expressed in adipose tissue [5].

Mouse model studies have shown that in the high-fat diet, VDR-knockout (VDR−/−) mice were prone to weight-gain resistance [19, 20]. It seems that overexpression of human VDR in adipocytes leads to a decrease in the energy expenditure and an increase in the body weight [9, 10]. Although, 1,25-dihydroxyvitamin D3 modulates adipogenic differentiation at several stages, there are significant variations in different cell types [20, 21].

It is assumed that expression of the main adipogenic genes related to early and late stages of differentiation including peroxisome proliferator-activated receptor-gamma (PPARγ), CCAAT-enhancer-binding protein-α (C/EBPα), lipoprotein lipase (LPL), and adipocyte protein 2 (aP2) is blocked by 1,25-dihydroxyvitamin D3, dose-dependently [20–25]. Additionally, 1,25-dihydroxyvitamin D3 has been shown to suppress adipocyte differentiation in the early stages by inhibiting CCAAT-enhancer-binding protein-β (C/EBPβ) expression, indirectly downregulating PPARγ and C/EBPα expression in 3T3-L1 cells [26]. Furthermore, the presence of vitamin D response element in the promoter region of Insig-2 has highlighted another novel mechanism regarding the inhibitory effects of 1,25-dihydroxyvitamin D3 [27].

There is limited evidence on mesenchymal stem cells derived from human adipose and the mechanisms by which, 1,25-dihydroxyvitamin D3 influences adipogenesis and energy balance. Therefore, in the present research, the mechanisms underlying outcome of 1,25-dihydroxyvitamin D3 action on expression of adipogenic genes in mesenchymal stem cells derived from human adipose were investigated.

## Materials and methods

### Cell culture and differentiation

Human adipose-derived mesenchymal stem cells (hASCs) were obtained from Human Cell Bank of the Iranian Biological Resource Center Laboratory (Tehran, Iran). The hASCs were obtained from subcutaneous abdominal adipose tissue of 5 premenopausal female donors with a mean age of 37 years old (with an age range from 28 to 39 years old) and a mean body mass index(BMI) of 26.2 [range: 24.5–29.3] through optional liposuction procedures. None of the volunteers had any type of endocrine disorders and none of them were taking any medication or had a family history of metabolic syndrome.

The hASCs were characterized based on their plastic and fibroblast-like morphology, capability to form colony-forming units (CFUs), expression of cell surface markers (cluster of differentiation(CD) antigens including CD44^+^, CD90^+^, CD105^+^, CD166^+^, CD34^-^, CD45^-^, and CD11b^-^) ,and the ability to differentiate into either osteoblasts or adipocytes as described previously [28].

Dulbecco’s modified Eagle’s medium (DMEM) supplemented with fetal bovine serum (FBS) 10%, glutamine 2%, 100 IU/ml of penicillin,and 100 IU/ml of streptomycin was used as a growth medium, which was incubated at 37°C and under 5% humidified CO_2_,and then was replaced every 2 days. For induction of differentiation into mature adipocytes 48 h post-confluence, the cells from passages of 4–5 were washed thoroughly using phosphate-buffered saline (PBS) and were seeded at seeding density of 5.04231 cells/ml. An amount of cells was pre-optimized in adipocyte differentiation medium (Gibco, UK) containing 0.5mM 3-isobutyl-3-methylxanthine (IBMX), 1mM dexamethasone, and 5mg/ml of human insulin. At the time of induction of differentiation of mesenchymal pre-adipocytes, 1,25-dihydroxyvitamin D3 was diluted in ethanol (vehicle) to obtain appropriate concentrations of 10^−10^ and 10^−8^ M, which were added to the medium and then, was kept for 14 days. Wells were divided into three experimental groups with at least three parallel wells in each group: (1) 10^−10^ M of 1,25-dihydroxyvitamin D3 with induction; (2) 10^−8^ M of 1,25-dihydroxyvitamin D3 with induction; (3) and control with induction. After a week, medium was replaced with an adipocyte maintenance medium (Gibco, UK) and it was cultured for another 7 days. Treatment compounds were added at same concentrations when medium was changed. All of these mixtures were also exchanged in the control wells. Cells were harvested to assess the genes related to adipogenesis at 1and 3 h and on 1, 3, 6, and 14 days during differentiation. Unless otherwise noted, all the chemical materials were purchased from Sigma-Aldrich Company (USA).

### RNA isolation and quantitative real-time PCR

Total RNA was isolated from the treated differentiating cells as described at several time points using the TRIzol reagent (Sigma-Aldrich Company, USA) according to the manufacturer’s instruction, then RNA was reverse transcribed into cDNA using the SuperScript II Reverse Transcriptase Kit (Invitrogen Company, USA) following the manufacturer’s protocol. Quantitative polymerase chain reaction (qPCR) analysis was performed using the StepOnePlus Real-Time PCR System (Applied Biosystems Company, USA) and SYBR Premix Ex Taq II, Tli RNaseH Plus reagent (Takara Company, Japan). Primer pairs were designed for PPARγ, C/EBPα, C/EBPβ, SREBP1c, FASN, LPL, and Insulin induced gene 2 (INSIG2) using the Primer-BLAST software (national center for biotechnology information (NCBI), USA). The mRNA levels were normalized to glyceraldehyde-3-phosphate dehydrogenase (GAPDH) and fold changes in gene expression were calculated by the 2^−ΔΔCt method^. The primer pairs for each gene target are presented in Table 1.

**Table 1:** The name and sequence of the primers, the sizes, and annealing temperatures for each pair

Gene	Size (bp)	Sequence (5′→3′)	Annealing temperature (°C)
GAPDH	113	F:CATGAGAAGTATGACAACAGCCT R:AGTCCTTCCACGATACCAAAGT	58
PPARƔ	80	F:CAGAAATGCCTTGCAGTGGG R:AACAGCTTCTCCTTCTCGGC	59
CEBPα	94	F:TATAGGCTGGGCTTCCCCTT R:AGCTTTCTGGTGTGACTCGG	60
CEBPβ	154	F:TTTGTCCAAACCAACCGCAC R:GCATCAACTTCGAAACCGGC	59
SREBP1c	117	F:TCTCAGTCCCCTGGTCTCTG R:ATAGGCAGCTTCTCCGCATC	59
INSIG2	114	F:AGTGGTCCAGTGTAATGCGG R:TGGATAGTGCAGCCAGTGTG	60
LPL	137	F:GCTCAGGAGCATTACCCAGTGTC R:GCTCCAAGGCTGTATCCCAAGA	63
FASN	107	F:ATTCTGCCATAAGCCCTGTC R:CTGTGTACTCCTTCCCTTCTTG	57

GAPDH: Glyceraldehyde-3-phosphate dehydrogenase; PPARγ: Peroxisome proliferator-activated receptor-gamma; C/EBPα: CCAAT-enhancer-binding protein-alpha; C/EBPβ: CCAAT-enhancer-binding protein- beta; SREBP1c: Sterol regulatory element-binding protein-1c; INSIG2: Insulin induced gene-2; FASN: Fatty acid synthase; LPL: lipoprotein lipase

## Oil Red O staining

Following 6 or 14 days of culture, adipocytes were washed three times using ice cold PBS and were fixed using paraformaldehyde 4% for 30 min. After fixation, cells were washed three times and were stained with Oil Red O solution (ORO) for 15 min at room temperature. Again, cells were washed three times by PBS to remove unbound staining in order to detect neutral lipid vacuoles. ORO-stained adipocytes were observed under a microscope (Olympus Company, Tokyo, Japan) and digital images were captured at 100× of magnification.

## Protein assay

For determining protein concentration, the plated cells were lysed in buffer containing 50mM Tris, 150mM sodium chloride (NaCl), IGEPAL 1%, 5mM ethylenediaminetetraacetic acid (EDTA) (Sigma-Aldrich Company,USA), and protease inhibitor cocktail (Roche Diagnostics, Laval, QC, Canada) and were centrifuged for collection of lysate. Then, enzyme-linked immunosorbent assay (ELISA) kits (ZellBio GmbH, Ulm, Germany) were used for assessment of FABP4, GLUT4, and VDR proteins in the tissue using spectrophotometer (Epoch Model, BioTek, Vermont, USA) on days 6 and 14 by intra-assay coefficients of variation (CVs) of 5.5, 5.8, 6.1, and 5.9, respectively.

### Statistical analysis

Data were expressed as mean ± standard deviation (SD). The mRNA expressions were determined by analysis of variance (ANOVA) and the Repeated-Measures test using SPSS software version 25 for Windows (standard version; SPSS Inc., Chicago, IL, USA) and GraphPad software (GraphPad Prism 8.01 Software) was applied to draw the graphs. Kruskal-Wallis test along with Dunn's test was used to compare level of protein expressions between the groups. Two-tailed p-values of <0.05 were considered as statistically significant.

## Results

**Fig. 1:**
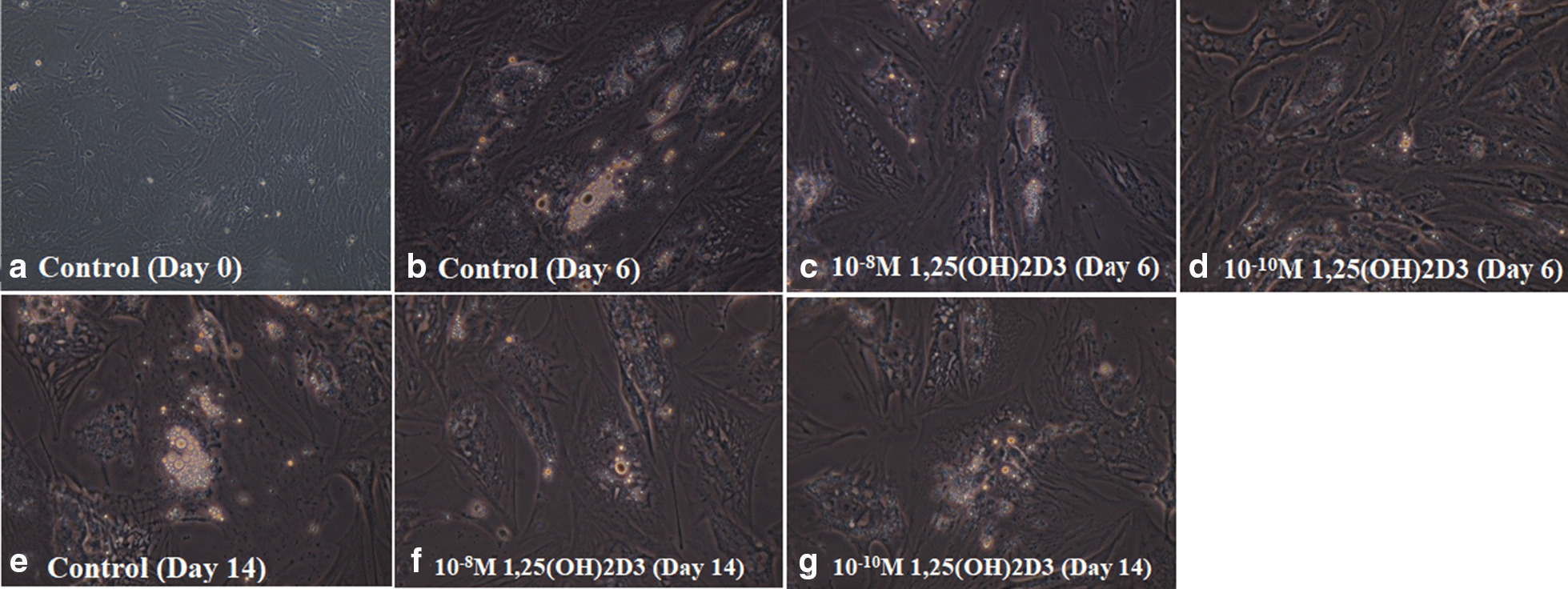
Oil Red O staining of human adipose-derived mesenchymal stem cells. Phase contrast image of adipocytes were taken by microscope (Olympus, Tokyo, Japan) and digital images were captured at ×100 magnification. Following 14 days of treatment with 1,25(OH)2D3 showed a significant decreased in relative lipid vacuole staining compared with control group

Morphology of hASCs and lipid accumulation were depicted through differentiation (Fig. 1).

### In human mesenchymal stem cells, 10^−10^ M 1,25-dihydroxyvitamin D3 inhibited adipogenesis While 10^−8^ M 1,25-dihydroxyvitamin D3 had stimulating effect

**Fig. 2:**
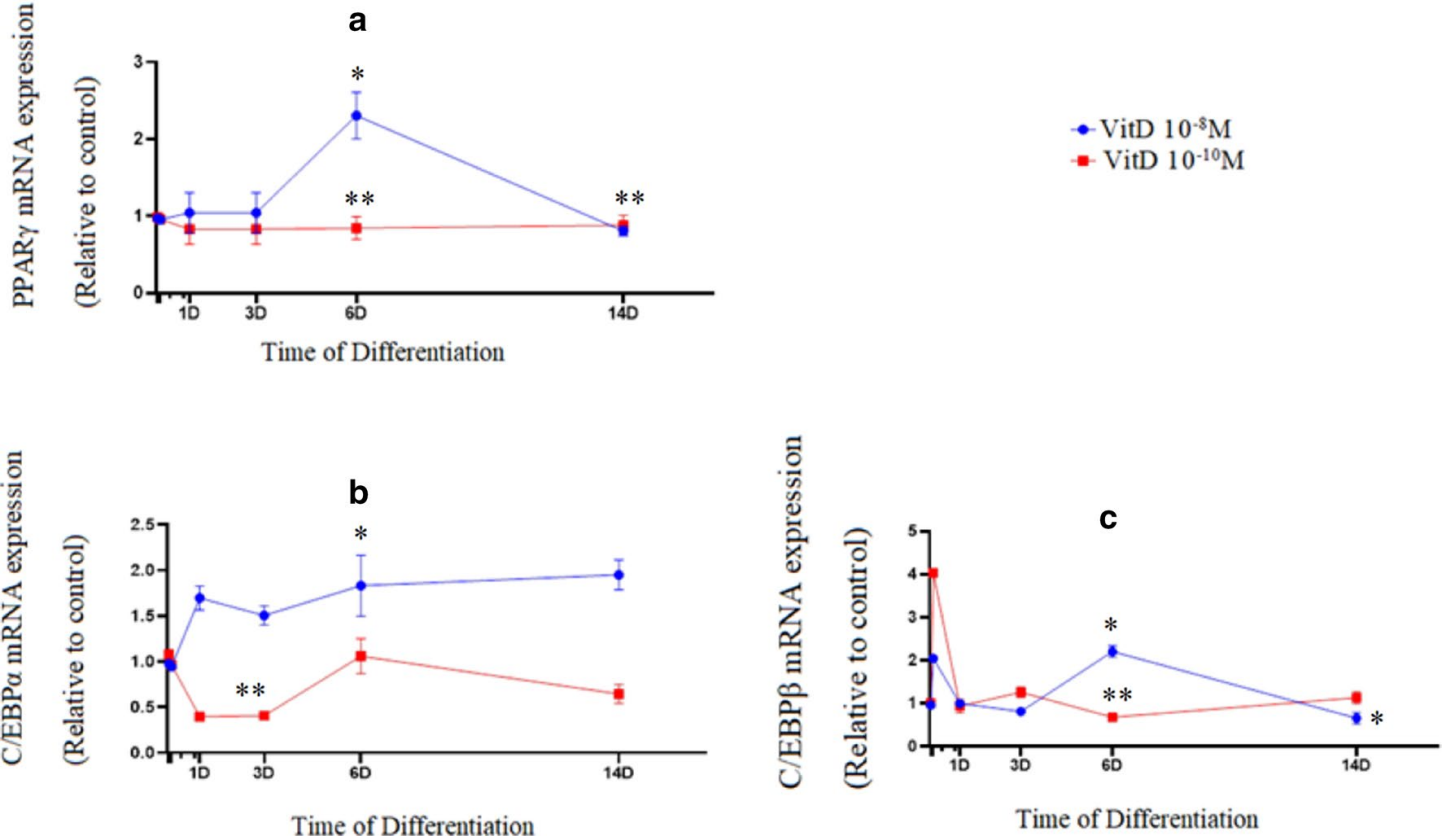
1,25(OH)2D3 differentially regulates the mRNA expression of adipogenic marker genes during adipogenesis. mRNA expression of PPARγ (**a**), C/EBPα (**b**) and C/EBPβ (**c**) in 1,25(OH)2D3 (10^−8^ M or 10^−10^ M) groups during adipogenic differentiation. The relative qPCR values were corrected to GAPDH expression levels and normalized with respect to controls on each time. The mRNA levels are expressed as the fold increase relative to the control, and values given are the mean ± SD with 95% CIs for three independent plates. *P < 0.05 (10^−8^ M 1,25(OH)2D3), **P < 0.05 (10^−10^ M 1,25(OH)2D3) vs. control

For investigating molecular mechanism of 1,25-Dihydroxyvitamin D3, qPCR was carried out for C/EBPα, C/EBPβ, FASN, LPL, PPARγ, SREBP1c ,and INSIG2 throughout differentiation. The anti-lipogenic outcome of 1,25-Dihydroxyvitamin D3 through adipogenesis was accompanied by alterations in the expression of adipogenic markers involved in metabolism of adipose tissue. Results showed upregulation of PPARγ (Fig. 2a), as the master transcriptional regulator of adipogenesis, through treatment with 1,25-Dihydroxyvitamin D3 at a concentration of 10^−8^M on day 6 (*P*=0.015). Moreover, mRNA expression of PPARγ did not change significantly throughout differentiation by treatment with 1,25-Dihydroxyvitamin D3 at a concentration of 10^−10^ M. Expression of C/EBPα (*P*=0.01) was downregulated during treatment with 1,25-Dihydroxyvitamin D3 (10^10^ M) on day 3. However, expression of C/EBPα mRNA was augmented (*P*=0.044) during differentiation by treatment with 1,25-Dihydroxyvitamin D3 at a concentration of 10^−8^M, (Fig. 2b) on day 6.

After observing a peak on day 6 (*P*=0.003), mRNA expression of C/EBPβ was significantly downregulated by treatment with 1,25-Dihydroxyvitamin D3 at a concentration of 10^−8^M on day 14 (*P*=0.008) and there was a fluctuation in C/EBPβ mRNA expression by treatment with 1,25-Dihydroxyvitamin D3 at a concentration of 10^−10^M along with downregulation on day 6 (*P*<0.001) throughout differentiation (Fig. 2c).

### 10^−8^ M of 1,25-dihydroxyvitamin D3 augmented expression of lipogenic enzymes

**Fig. 3:**
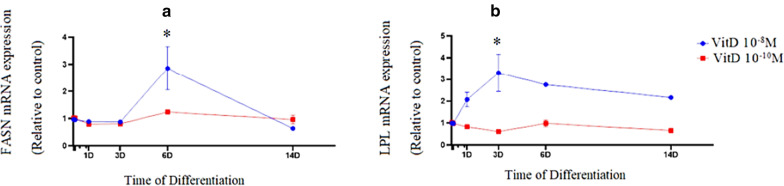
1,25(OH)2D3 increased mRNA expression of FASN and LPL in newly-differentiated adipocytes at high concentrations. mRNA expression of FASN (**a**) and LPL (**b**) in 1,25(OH)2D3 groups during adipogenic differentiation.The relative qPCR values were corrected to GAPDH expression levels and normalized with respect to controls on each time. The mRNA levels are expressed as the fold increase relative to the control, and values given are the mean ± SD with 95% CIs for three independent plates. *p < 0.05 (10^−8^ M 1,25(OH)2D3 vs. control)

**Fig. 4:**
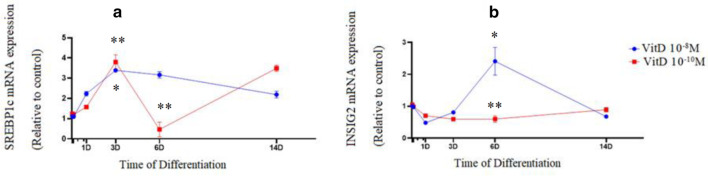
1,25(OH)2D3 differentially regulates the mRNA expression of adipogenic marker genes during adipogenesis. mRNA expression of SREBP1c (**a**) and INSIG2 (**b**) in 1,25(OH)2D3 groups during adipogenic differentiation.The relative qPCR values were corrected to GAPDH expression levels and normalized with respect to controls on each time. The mRNA levels are expressed as the fold increase relative to the control, and values given are the mean ± SD with 95% CIs for three independent plates. *p < 0.05 (10^−8^ M 1,25(OH)2D3), **p < 0.05 (10^−10^ M 1,25(OH)2D3) vs. control

During adipogenic differentiation, mRNA expression of FASN, as a marker of de novo lipogenesis did not change significantly by treatment with 1,25-Dihydroxyvitamin D3 at a concentration of 10^−10^ M. Expression of FASN (*P*=0.049) was upregulated by treatment with 1,25-Dihydroxyvitamin D3 at a concentration of 10^−8^ M on day 6 (Fig. 3(a)). There was no change in mRNA expression of LPL, as a late marker of adipogenesis, throughout treatment with 1,25-Dihydroxyvitamin D3 at a concentration of 10^−10^ M. On the other hand, mRNA expression of LPL was augmented (*P*=0.044) through treatment with 1,25-Dihydroxyvitamin D3 at a concentration of 10^−8^ M with a peak observed on day 3 (Fig. 3b). Upregulation of PPARγ expression was accompanied by overexpression of SREBP1c mRNA by treatment with 1,25-Dihydroxyvitamin D3 at a concentration of 10^−8^ M on day 3 (*P*<0.001). A fluctuation in mRNA expression of SREBP1c was observed with a downregulation by treatment with 1,25-Dihydroxyvitamin D3 at a concentration of 10^−10^ M , on day 6 (*P*<0.001) (Fig. 4a). Since, 1,25-Dihydroxyvitamin D3 upregulated expression of PPAR-γ and SREBP-1, expression of INSIG2, as a moderator of PPAR-γ and SREBP-1c was also investigated. Following an overexpression of INSIG2 on day 3, mRNA expression of INSIG2 was significantly downregulated by treatment with 1,25-Dihydroxyvitamin D3 at a concentration of 10^−10^ M on day 6. Overexpression of INSIG2 mRNA was observed in the group treated with 1,25 Dihydroxyvitamin D3 at a concentration of 10^-8^M on day 6 (P=0.022) (Fig. 4(b)).

### VDR, GLUT4, and FABP4 proteins were expressed in human adipose-derived mesenchymal stem cells

For understanding the important role of VDR regarding modification of adipogenesis by 1,25-Dihydroxyvitamin D3, expression of VDR protein in hASCs was investigated at specific time intervals after differentiation process was initiated. Results showed that protein level of VDR was lower than that of the control group during differentiation process (Table 2). Following 14 days of treatment with 1,25-Dihydroxyvitamin D3, protein level of GLUT4 and FABP4 was also less than that of the control group (Table 2).

**Table 2:** Comparison of protein expression in 1,25-Dihydroxyvitamin D3 groups vs. control

Proteins	Time	Group	Mean§	Standard Deviation	Result
FABP4	Day 6	Control	0.27	0.03	X = 8.76 *df* = 2 *P* value = 0.012*
		10^−8^MVitD	0.17	0.007	
		10^−10^ M VitD	0.14	0.03	
	Day 14	Control	0.32	0.02	X = 7. 26 *df* = 2 *P* value = 0.026*
		10^−8^ M VitD	0.20	0.04	
		10^−10^ M VitD	0.24	0.05	
GLUT4	Day 6	Control	0.17	0.03	X = 8.76 *df* = 2 *P* value = 0.012*
		10^−8^ M VitD	0.12	0.02	
		10^−10^ M VitD	0.08	0.02	
	Day 14	Control	0.20	0.01	X = 7. 26 *df* = 2 *P* value = 0.026*
		10^−8^ M VitD	0.20	0.03	
		10^−10^ M VitD	0.15	0.03	
VDR	Day 6	Control	0.69	0.45	X = 4.19 *df* = 2 *P* value = 0.012*
		10^−8^ M VitD	0.44	0.02	
		10^−10^ M VitD	0.35	0.08	
	Day 14	Control	0.80	0.05	X = 7. 26 *df* = 2 *P* value = 0.026*
		10^−8^ M VitD	0.50	0.09	
		10^−10^ M VitD	0.60	0.13	

FABP4: Fatty acid binding proteins-4; GLUT4: Glucose transporter-4; VDR: Vitamin D receptor § ng/mg total protein

^*^Mean values were significantly different between the groups (P < 0.05)

## Discussion

Our results provided unique insights into molecular modifications at each phase influencing adipogenesis in human mesenchymal stem cells by 1,25-Dihydroxyvitamin D3 treatment.

Most of our knowledge about adipocyte biology and adipogenesis has been achieved through cell culture model systems. The main types of cell model are human or animal-isolated cells directly obtained from stromal vascular adipose tissues [29, 30], multipotent fibroblastic cell lines that are not committed with adipogenic potential, and the established preadipocyte cell lines, undergone commitment to the adipose lineage [31].

For comprehending the role of vitamin D in adipogenesis, epidemiological studies have shown that 25-hydroxyvitamin D3 was negatively associated with body fat content and BMI [32, 33] and 1,25-Dihydroxyvitamin D3 presented unpredictable outcomes at higher concentrations in the obese people. However, further studies have indicated lower 1,25-Dihydroxyvitamin D3 levels in the obese population compared to non-obese population [32]. In a double-blind randomized clinical trial, we found that improving 25(OH) D serum concentrations by vitamin D3 supplementation is associated with lower body fat mass [34].

Since, Vitamin D influences differentiation of common progenitor mesenchymal stem cells in bone marrow to preosteoblasts, it is assumed that 1,25-Dihydroxyvitamin D3 can play a prominent role in differentiation of mesenchymal stem cells to adipocytes through unknown molecular pathways [19]. The inhibitory effect of 1,25-Dihydroxyvitamin D3 on adipocyte differentiation was first reported several years ago when in a study, lipid accumulation was reduced by half in 3T3-L1 and ST-13 preadipocytes compared to the control group [35, 36], suggesting that 1,25-Dihydroxyvitamin D3 prohibits adipogenic differentiation via receptor-mediated pathways [35]. On the other hand, according to the literature, 1,25-Dihydroxyvitamin D3 stimulates FAS in human adipocytes along with suppression of uncoupling protein 2 (UCP-2) and leptin [23, 37].

1,25-Dihydroxyvitamin D3 and VDR have been reported to be involved in regulation of adipogenesis. The evidence shows that 1,25-Dihydroxyvitamin D3 suppresses differentiation of 3T3-L1 cells in a dose-dependent manner,which is consistent with its inhibitory effect on transcription of adipogenic markers [2]. In contrast, it has been shown that 1,25-Dihydroxyvitamin D3 possibly stimulates differentiation of human preadipocytes by sustaining overexpression level of key adipogenic transcription factors [13]. C/EBPα and PPARγ have been introduced as the target of other adipogenic inhibitors [25] and overexpression of C/EBPβ is an initial incident that is vital for mitotic clonal expansion [26]. According to our findings, in the hASCs model, 1,25-Dihydroxyvitamin D3 (10^−10^ M) prohibits adipogenesis possibly by repressing mRNA expression of C/EBPβ, PPARγ,and adipogenic enzymes including FASN and LPL, together with stabilizing VDR protein level throughout late stage of differentiation. While, mRNA expression of these adipogenic markers was upregulated in the cells treated with 1,25-Dihydroxyvitamin D3 at a concentration of 10^−8^M. It was found that 1,25-Dihydroxyvitamin D3 influenced expression of C/EBPα and C/EBPβ mRNA through adipocyte differentiation. 1,25-Dihydroxyvitamin D3 (10^−10^ M) inhibited expression of C/EBPα and PPARγ and antagonized transcriptional action of PPARγ. Suppression of C/EBPα and PPARγ by 1,25-Dihydroxyvitamin D3 may inhibit differentiation of 3T3-L1 cells [23]. Since, retinoid X receptor (RXR) is a common heterodimeric partner of both VDR and PPARγ, 1,25-Dihydroxyvitamin D3 represses transcriptional action of PPARγ by emulating for inadequate quantity of RXR through VDR. In the key phase of adipogenic process, when 1,25-Dihydroxyvitamin D3 binds and activates VDR, it may sequester RXR from PPARγ [23, 27].

In the case of VDR overexpression, abundant amount of VDR in the cells may sequester RXR without ligand activation and overexpression of RXR can actually prevent sequestering action of VDR [23, 27]. Interestingly, expression of INSIG2 has been found to inhibit differentiation of preadipocytes and to control lipogenesis in mature adipocytes, probably shutting off extra synthesis of triglyceride for tissues, in which SREBPs play critical roles [38, 39]. In the present experiment, 1,25-Dihydroxyvitamin D3 prompted INSIG2 expression at the concentration of 10^−8^ M. Given the fact that SREBP1c is a potent marker of adipogenesis, our findings suggest that the inhibitory effect of 1,25-Dihydroxyvitamin D3 in adipogenesis may also be associated with overexpression of INSIG2 in early stages of lipogenesis. Expression level of SREBP-1c, as a proadipogenic marker was upregulated significantly in the cells treated with 1,25-Dihydroxyvitamin D3 at the concentration of 10^−8^M. Further studies are also warranted to conclude whether the augmented INSIG2 expression upon 1,25-Dihydroxyvitamin D3 treatment leads to reduction of SREBP1c levels in 3T3-L1 preadipocytes and inhibit adipogenesis [40]. In line with our results indicating the stimulating effect of 1,25-dihydroxyvitamin D (10^−8^ M) on expression of LPL mRNA, Nimitphong et al., showed an increase in the LPL expression in 3T3-L1 preadipocyte [13]. Kang et al., also proposed that reduction of body weight and fat deposition after treatment with vitamin D3 is possibly attributed to modulation of lipogenic enzymes, FAS, stearoyl-CoA desaturase 1 (SCD1),and acetyl-CoA carboxylase 1 (ACC1) in adipocytes of the pregnant rats [40]. Studies have shown the pro-adipogenic effects of 1,25-Dihydroxyvitamin D3 in human preadipocytes in contrast with its anti-adipogenic effect in the 3T3-L1, as the commonly used preadipocyte cell line. Treatment of 3T3-L1 with 1,25-Dihydroxyvitamin D3 through early induction stage is important for its inhibitory effect as confirmed in the current study [13, 23]. Adipogenesis was not induced when 1,25(OH)2D3 was added through the 3rd-induction phase.

Whereas, adding 1,25-Dihydroxyvitamin D3 within maturation period similarly induces adipogenesis as the continuous treatment [42]. Since, FABP4 and GLUT4 are essential for adipocyte function, our findings demonstrated that protein level of FABP4 and GLUT4 was upregulated by 1,25-Dihydroxyvitamin D3 (Table 2). However, expression level of FABP and GLUT4 proteins was higher in the control group compared to the groups treated with 1,25-Dihydroxyvitamin D3. In hASCs, expression of FABP4 was increased in adipogenic medium compared to basic medium. In the cells treated with 1,25-Dihydroxyvitamin D3, expression of FABP4 was further upregulated [20]. Additionally, Nemetphong et al., demonstrated that 1,25-Dihydroxyvitamin D3 augmented expression level of FABP4 protein, dose-dependently [13]. Regulation of lipid and glucose metabolism is a key function of adipocytes, which depends on uptake of glucose by GLUT4, as the major insulin-dependent glucose transporter in skeletal muscle, heart, and adipocyte tissues. In the humans and rodents, expression of GLUT4 is reduced in adipocytes due to obesity or type 2 diabetes attributing to pathogenesis of insulin resistance and type 2 diabetes [43]. It has been stated that PPARγ activates C/EBPα gene promoter through a positive feedback and then, induces expression of the genes involved in insulin sensitivity, lipogenesis, and lipolysis including GLUT4 and FABP4 [44].

## Conclusion

Results of the present study indicated that treatment of human mesenchymal stem cells with 1,25-Dihydroxyvitamin D3 at a concentration of 10^-8^M enhanced expression of adipogenic-related genes including PPAR-γ, FASN, and LPL.

One of the limitations of the present study was that during morphological assessment of the differentiated cells, despite observing more lipid vacuoles in the control group compared to the groups treated with 1,25-Dihydroxyvitamin D3, lipid accumulation was not quantitatively measured. The genomic and non-genomic pathways controlling vitamin D endocrine system in human adipocytes are also suggested to be further investigated.

## Data Availability

Not applicable.

## Abbreviations

ACC1: Acetyl-CoA carboxylase 1
ANOVA: Analysis of variance
aP2: Adipocyte protein 2
BMI: Body mass index
CD: Cluster of differentiation
C/EBPα: CCAAT-enhancer-binding protein-α
C/EBPβ: CCAAT-enhancer-binding protein-β
CFUs: Colony-forming units
DMEM: Dulbecco’s modified Eagle’s medium
EDTA: Ethylenediaminetetraacetic acid
ELISA: Enzyme-linked immunosorbent assay
FABP4: Fatty acid binding protein-4
FASN: Fatty acid synthase
FBS: Fetal bovine serum
GAPDH: Glyceraldehyde-3-phosphate dehydrogenase
GLUT4: Glucose trasporter-4
hASCs: Human adipose-derived mesenchymal stem cells
IBMX: 3-Isobutyl-3-methylxanthine
INSIG2: Insulin induced gene 2
LPL: Lipoprotein lipase
NaCl: Sodium chloride
NCBI: National Center for Biotechnology Information
ORO: Oil Red O solution
PBS: Phosphate-buffered saline
PCR: Quantitative polymerase chain reaction
PPARγ: Peroxisome proliferator-activated receptor-gamma
RXR: Retinoid X receptor
SCD1: Stearoyl-CoA desaturase 1
SD: Standard deviation
SREBP1c: Sterol regulatory element-binding protein-1c
VDR: Vitamin D receptor
VDR^−/−^: VDR-knockout
vitamin D3: Cholecalciferol
UVB: Ultraviolet B
UCP-2: Uncoupling protein 2
25(OH)D: 25-Hidroxyvitamin D3
1,25(OH)_2_D: 1,25-Dihydroxy-vitamin D

## References

[1] Heymsfield SB, Wadden TA (2017). Mechanisms, pathophysiology, and management of obesity. N Engl J Med.

[2] Gadde KM, Martin CK, Berthoud HR, Heymsfield SB (2018). Obesity: pathophysiology and management. J Am Coll Cardiol.

[3] Tremmel M, Gerdtham UG, Nilsson PM, Saha S (2017). Economic burden of obesity: a systematic literature review. Int J Environ Res Public Health.

[4] GBD (2015). Obesity Collaborators (2017) Health effects of overweight and obesity in 195 countries over 25 years. N Engl J Med.

[5] Walsh JS, Bowles S, Evans AL (2017). Vitamin D in obesity. Curr Opin Endocrinol Diabetes Obesity.

[6] Pourshahidi LK (2015). Vitamin D and obesity: current perspectives and future directions. Proc Nutr Soc.

[7] Wimalawansa SJ (2018). Associations of vitamin D with insulin resistance, obesity, type 2 diabetes, and metabolic syndrome. J Steroid Biochem Mol Biol.

[8] Wimalawansa SJ (2018). Non-musculoskeletal benefits of vitamin D. J Steroid Biochem Mol Biol.

[9] Mutt SJ, Hyppönen E, Saarnio J, Järvelin MR, Herzig KH (2014). Vitamin D and adipose tissue—more than storage. Front Physiol.

[10] Vinh Quốc Lương K, Nguyễn LT (2013). The beneficial role of vitamin D in obesity: possible genetic and cell signaling mechanisms. Nutr J.

[11] Bouillon R, Carmeliet G, Lieben L, Watanabe M, Perino A, Auwerx J (2014). Vitamin D and energy homeostasis—of mice and men. Nat Rev Endocrinol.

[12] Abbas MA (2017). Physiological functions of Vitamin D in adipose tissue. J Steroid Biochem Mol Biol.

[13] Nimitphong H, Holick MF, Fried SK, Lee MJ (2012). 25-hydroxyvitamin D3 and 1,25- dihydroxyvitamin D3 promote the differentiation of human subcutaneous preadipocytes. PLoS ONE.

[14] Pannu PK, Zhao Y, Soares MJ (2016). Reductions in body weight and percent fat mass increase the vitamin D status of obese subjects: a systematic review and metaregression analysis. Nutr Res.

[15] Rejnmark L, Bislev LS, Cashman KD, Eiríksdottir G, Gaksch M, Grübler M (2017). Non-skeletal health effects of vitamin D supplementation: a systematic review on findings from meta-analyses summarizing trial data. PLoS ONE.

[16] Landrier JF, Karkeni E, Marcotorchino J, Bonnet L, Tourniaire F (2016). Vitamin D modulates adipose tissue biology: Possible consequences for obesity?. Proc Nutr Soc.

[17] Ching S, Kashinkunti S, Niehaus MD, Zinser GM (2011). Mammary adipocytes bioactivate 25-hydroxyvitamin D3 and signal via vitamin D3 receptor, modulating mammary epithelial cell growth. J Cell Biochem.

[18] Trayhurn P, O'Hara A, Bing C (2011). Interrogation of microarray datasets indicates that macrophage-secreted factors stimulate the expression of genes associated with vitamin D metabolism (VDR and CYP27B1) in human adipocytes. Adipobiology.

[19] Ding C, Gao D, Wilding J, Trayhurn P, Bing C (2012). Vitamin D signaling in adipose tissue. Br J Nutr.

[20] Narvaez CJ, Matthews D, Broun E, Chan M, Welsh J (2009). Lean phenotype and resistance to diet-induced obesity in vitamin D receptor knockout mice correlates with induction of uncoupling protein-1 in white adipose tissue. Endocrinology.

[21] Ruiz-Ojeda FJ, Anguita-Ruiz A, Leis R, Aguilera CM (2018). Genetic factors and molecular mechanisms of Vitamin D and obesity relationship. Ann Nutr Metab.

[22] Wong KE, Kong J, Zhang W, Szeto FL, Ye H, Deb DK et al. Targeted expression of human vitamin D receptor in adipocytes decreases energy expenditure and induces obesity inmice. J Biol Chem jbc-M111 (2018). 10.1074/jbc.M111.257568 PMC3190828 21840998

[23] Kong J, Li YC (2006). Molecular mechanism of 1, 25-dihydroxyvitamin D3 inhibition of adipogenesis in 3T3-L1 cells. Am J Physiol Endocrinol Metab.

[24] Lee S, Lee DK, Choi E, Lee JW (2005). Identification of a functional vitamin D response element in the murine Insig-2 promoter and its potential role in the differentiation of 3T3-L1 preadipocytes. Mol Endocrinol.

[25] Neal JW, Clipstone NA (2002). Calcineurin mediates the calcium-dependent inhibition of adipocyte differentiation in 3T3-L1 cells. J Biol Chem.

[26] Tang QQ, Otto TC, Lane MD (2003). CCAAT/enhancer-binding protein β is required for mitotic clonal expansion during adipogenesis. Proc Natl Acad Sci USA.

[27] Demay MB (2006). Mechanism of vitamin D receptor action. Ann N Y Acad Sci.

[28] Bourin P, Bunnell BA, Casteilla L, Dominici M, Katz AJ, March KL (2013). Stromal cells from the adipose tissue-derived stromal vascular fraction and culture expanded adipose tissue-derived stromal/stem cells: a joint statement of the International Federation for Adipose Therapeutics and Science (IFATS) and the International Society for Cellular Therapy (ISCT). Cytotherapy.

[29] Ntambi JM, Young-Cheul K (2000). Adipocyte differentiation and gene expression. J Nutr.

[30] Otto TC, Lane MD (2005). Adipose development: from stem cell to adipocyte. Crit Rev Biochem Mol Biol.

[31] Cristancho AG, Lazar MA (2011). Forming functional fat: a growing understanding of adipocyte differentiation. Nat Rev Mol Cell Biol.

[32] Parikh SJ, Edelman M, Uwaifo GI, Freedman RJ, Semega-Janneh M, Reynolds J (2004). The relationship between obesity and serum 1, 25-dihydroxy vitamin D concentrations in healthy adults. J Clin Endocrinol Metab.

[33] Yanoff LB, Parikh SJ, Spitalnik A, Denkinger B, Sebring NG, Slaughter P (2006). The prevalence of hypovitaminosis D and secondary hyperparathyroidism in obese Black Americans. Clin Endocrinol.

[34] Salehpour A, Hosseinpanah F, Shidfar F, Vafa M, Razaghi M, Dehghani S (2012). A 12-week double-blind randomized clinical trial of vitamin D 3 supplementation on body fat mass in healthy overweight and obese women. Nutr J.

[35] Ishida Y, Taniguchi H, Baba S (1988). Possible involvement of 1α, 25-dihydroxyvitamine D3 in proliferation and differentiation of 3T3-L1 cells. Biochem Biophys Res Commun.

[36] Sato M, Hiragun A (1988). Demonstration of 1α, 25-dihydroxyvitamin D3 receptor-like molecule in ST 13 and 3T3 L1 preadipocytes and its inhibitory effects on preadipocyte differentiation. J Cell Physiol.

[37] Sun X, Zemel MB (2004). Role of uncoupling protein 2 (UCP2) expression and 1α, 25-dihydroxyvitamin D3 in modulating adipocyte apoptosis. FASEB J.

[38] Li J, Takaishi K, Cook W, McCorkle SK, Unger RH (2003). Insig-1 “brakes” lipogenesis in adipocytes and inhibits differentiation of preadipocytes. Proc Natl Acad Sci USA.

[39] Gascon-Barré M, Demers C, Mirshahi A, Néron S, Zalzal S, Nanci A (2003). The normal liver harbors the vitamin D nuclear receptor in nonparenchymal and biliary epithelial cells. Hepatology.

[40] Yabe D, Komuro R, Liang G, Goldstein JL, Brown MS (2003). Liver-specific mRNA for Insig- 2 down-regulated by insulin: implications for fatty acid synthesis. Proc Natl Acad Sci USA.

[41] Kang EJ, Lee JE, An SM, Lee JH, Kwon HS, Kim BC (2015). The effects of vitamin D3 on lipogenesis in the liver and adipose tissue of pregnant rats. Int J Mol Med.

[42] Wood RJ (2008). Vitamin D and adipogenesis: new molecular insights. Nutr Rev.

[43] Moraes-Vieira PM, Saghatelian A, Kahn BB (2016). GLUT4 expression in adipocytes regulates de novo lipogenesis and levels of a novel class of lipids with antidiabetic and anti-inflammatory effects. Diabetes.

[44] Lefterova MI, Zhang Y, Steger DJ, Schupp M, Schug J, Cristancho A, Feng D, Zhuo D, Stoeckert CJ, Liu XS, Lazar MA (2008). PPARγ and C/EBP factors orchestrate adipocyte biology via adjacent binding on a genome-wide scale. Genes Dev.

