# Correction to: 1,25-Dihydroxyvitamin D3 modulates adipogenesis of human adipose-derived mesenchymal stem cells dose-dependently

**DOI:** 10.1186/s12986-021-00568-x

**Published:** 2021-04-15

**Authors:** Amin Salehpour, Mehdi Hedayati, Farzad Shidfar, Asal Neshatbini Tehrani, Ali Asghar Farshad, Saeed Mohammadi

**Affiliations:** 1 Occupational Health Research Center, School of Public Health, Iran University of Medical Sciences, Tehran, Iran; 2 Cellular and Molecular Endocrine Research Center, Research Institute for Endocrine Sciences, Shahid Beheshti University of Medical Sciences, 2nd Floor, Number 24, Parvaneh Street, Yemen Street, Chamran Exp, Tehran, Iran; 3 Department of Nutrition, School of Paramedical, Ahvaz Jundishapur University of Medical Sciences, Ahvaz, Iran; 4 Department of Biostatics, School of Public Health, Iran University of Medical Sciences, Tehran, Iran

## Correction to: Nutr Metab (Lond) (2021) 18:29 10.1186/s12986-021-00561-4

Following publication of the original article [1], the authors identified an error in the affiliation of Dr. Mehdi Hedayati.

The incorrect affiliation: Cellular and Molecular Research Center, Research Institute for Endocrine Sciences, Shahid Beheshti University of Medical Sciences, 2nd Floor, Number 24, Parvaneh Street, Yemen Street, Chamran Exp, Tehran, Iran.

The correct affiliation: Cellular and Molecular Endocrine Research Center, Research Institute for Endocrine Sciences, Shahid Beheshti University of Medical Sciences, 2nd Floor, Number 24, Parvaneh Street, Yemen Street, Chamran Exp, Tehran, Iran.

The author group has been updated above and the original article [1] has been corrected.
